# Teaching of silver diamine fluoride for the management of dental caries and hypersensitivity – situation in the Southeast Asia dental schools

**DOI:** 10.1186/s12903-023-03502-0

**Published:** 2023-10-29

**Authors:** Shuyang He, Sicheng Wu, Duangporn Duangthip, Chun Hung Chu, Edward Chin Man Lo

**Affiliations:** 1 Applied Oral Sciences & Community Dental Care, Faculty of Dentistry, The University of Hong Kong, 34 Hospital Rd, Sai Wan Hong Kong, China; 2 Restorative Dental Sciences, Faculty of Dentistry, The Universitry of Hong Kong, 34 Hospital Rd, Hong Kong, Sai Wan China

**Keywords:** Silver diamine fluoride, Undergraduate teaching, Dental education, Southeast Asia

## Abstract

**Background:**

Using silver diamine fluoride (SDF) for caries management has raised dentists’ interests in Southeast Asia (SEA). However, information about the teaching of SDF in dental schools in SEA is limited. Therefore, this survey aimed to describe the extent to which SDF had been introduced into the education of undergraduate students in the dental schools in SEA.

**Methods:**

An online questionnaire survey was conducted on the duration, method, contents, and barriers regarding the teaching of SDF. Teachers in charge of undergraduate program in pediatric dentistry and those in community dentistry in all the 90 dental schools in SEA were approached and we required each department to reply once only. Descriptive statistics and Chi-square test were used to describe and assess the differences between the two departments in the teaching of SDF.

**Results:**

A total of 81 responses from the departments of 49 schools were received, giving a school-level response rate of 54% (49/90). SDF was taught in the undergraduate program in 86% (42/49) of the respondent schools, and 50% (21/42) of these schools had included SDF in the teaching for five or more years. Almost all (98%) of the departments taught SDF through lectures. Furthermore, 55% of them adopted SDF in clinical practice. Regarding the teaching content, the use of SDF for arresting cavitated caries lesion was the most commonly covered (82–97%), followed by for arresting early noncavitated lesions (69–82%), for preventing new caries development (66–79%) and for treating dental hypersensitivity (77%). There were variations in the post-treatment instruction taught. For the departments not teaching SDF, the most common reason (10/19, 53%) was that SDF was not available.

**Conclusion:**

SDF is covered in the undergraduate program in most of the dental schools in SEA. The use of SDF to arrest cavitated caries lesions in primary teeth is usually taught. However, other applications of SDF, such as for prevention of caries and treatment of dental hypersensitivity, are less commonly mentioned in the teaching.

**Supplementary Information:**

The online version contains supplementary material available at 10.1186/s12903-023-03502-0.

## Introduction

Dental caries is a prevalent disease worldwide affecting millions of people, especially the underprivileged populations and in developing countries [1, 2]. It significantly impacts on people’s oral and general health, as well as their quality of life. Southeast Asia (SEA) is a region consisting of 11 countries in which approximately 8.5% of the world’s population resides [3]. Most of the countries in SEA are developing countries. The median reported prevalence of caries experience and mean decayed, missing, filled teeth (dmft) score of 5- to 6-year-old children in this region were 79% and 5.1, respectively [4]. The prevalence of caries experience in the 15-year-old teenagers in SEA was 51% [5]. The age-standardized incidence rates of caries in the primary and permanent teeth of children in SEA were the highest and the second highest in the world, respectively [2].

Use of silver diamine fluoride (SDF) for caries management has recently been promoted in many countries worldwide. Its effectiveness in arresting cavitated caries lesions in both primary and permanent teeth has been proven [6, 7]. SDF has been increasingly used by dentists in many countries around the world because of its relatively low cost and ease of implementation [8, 9]. Furthermore, with the outbreak of the COVID-19 pandemic, there has been increasing concerns about the transmission of coronavirus through aerosols generated in restorative dental treatment procedures. Use of SDF is a good option for caries management because it is non-aerosol-generating and has low cross-infection risk [10].

There has been a significant increase in the number of clinical trials about SDF in SEA [11]. The Ministry of Public Health of Thailand has approved the use of SDF for treating coronal and root caries [12]. Moreover, a project conducted in Cambodia also included SDF as an essential treatment in universal oral health care [13].

Although ideally a standard dental curriculum should be designed for undergraduate students, it is unavoidable that considerable variations exist between schools in terms of the content and delivery of their dental education [14, 15]. SDF is used in clinical practice in many countries but the extent to which it is covered in the undergraduate teaching in dental schools is not known. Studies have shown that dental curriculum is sometimes disconnected from clinical evidence and practice [16, 17]. Whether the SDF-related teaching content changes with the update of clinical evidence has not been investigated. Understanding the current teaching is a prerequisite for curriculum development [18]. However, to the best of our knowledge, only a few studies in the United States and Canada have been conducted to investigate the teaching of SDF in dental schools. [17, 19–21] To date, no information about the teaching of SDF in dental schools in SEA is available.

Therefore, the aim of this study was: (1) to investigate the extent to which SDF had been introduced into the education of undergraduate dental students in SEA; (2) to describe the contents of the teaching of SDF; (3) to compare the teaching of SDF in pediatric dentistry and that in community dentistry; and (4) to investigate the reasons for not covering SDF in the undergraduate dental program.

## Method

This study was an online self-completion questionnaire survey. Ethics approval was obtained (IRB reference number: UW 22–572) from the Institutional Review Board of The University of Hong Kong in 2022.

### Survey participants

According to a comprehensive search on the internet, in mid-2022 there were a total of 90 dental schools/universities/colleges (hereinafter referred to as ‘dental schools’) in nine countries in SEA (Thailand, Malaysia, Indonesia, Singapore, Philippines, Myanmar, Laos, Cambodia, and Vietnam) which offered undergraduate dental program. Among these dental schools, 69 were members of the South East Asia Association for Dental Education (SEAADE). In this survey, there was no sampling and all 90 dental schools were invited to participate.

The deans of the dental schools and the heads of the departments (division, specialty) of pediatric dentistry and those of community dentistry (dental public health, preventive dentistry or equivalent) were approached. The department concerned was requested to choose one teacher in-charge (program director) from the department to complete the survey questionnaire, which means that up to two responses, one from each department, from each respondent school would be received. Their emails were obtained from the official websites of the dental school or other channels. The invitation email contained a brief introduction, informed consent and a link to the questionnaire. The introduction contained the purpose of the study and instructions on how to complete the questionnaire online. Reminder emails were sent to the potential participants if no response was received from them two weeks, four weeks, and seven weeks after the first email. The survey ended in October 2022.

### Survey instrument

The questionnaire, written in English and being anonymous, contained 15 questions (Appendix). The questions were adapted from those used in earlier studies [19, 21]. Most of the questions were at the department level. Only the fourth question in the questionnaire asked the respondent whether, to their best knowledge, at the school level (not limited to their own department), SDF was taught. A pre-test was conducted on teachers from two dental schools (one had covered SDF in its undergraduate curriculum and the other had not). Two teachers in each school completed the questionnaire and they were asked to provide suggestions to improve the wordings of the questions so as to enhance clarity and to avoid misunderstanding.

In the finalized questionnaire, the survey respondents were asked to provide the name of their dental school and their department. Information on the teaching of SDF (such as the amount of time allocated and the teaching methods used) and the content of the teaching was collected. If SDF was not included in their current undergraduate program, the respondents were asked to give the reasons and their plans. There was no skip option in the questionnaire and it could only be submitted when all questions had been completed. The questionnaire was designed and distributed using the web-based survey tool Qualtrics (Provo, UT, USA).

### Data analysis

The survey data were analyzed by the statistical software SPSS (SPSS Inc., Chicago, USA). Descriptive statistics were calculated for each question. Chi-square test were used to assess the differences between the two departments in the teaching of SDF. The statistical significance level was set at 0.05.

## Result

A total of 81 completed questionnaires (38 from pediatric dentistry department and 43 from community dentistry department) from 49 schools were received (response rate 54%, 49/90). The response rates by country and the proportion of the respondent schools covering SDF in the undergraduate teaching are presented in Table 1. There are seven dental schools that did not include SDF in any department.

**Table 1:** Survey response rate by country and the teaching of SDF in the respondent schools

Country	Survey response rate (No. of responses/No. of schools)	% respondent schools with teaching of SDF
Cambodia	100% (3/3)	100% (3/3)
Myanmar	100% (2/2)	100% (2/2)
Singapore	100% (1/1)	100% (1/1)
Thailand	87% (13/15)	100% (13/13)
Philippines	45% (9/20)	100% (9/9)
Indonesia	29% (8/27)	75% (6/8)
Vietnam	70% (7/10)	71% (5/7)
Malaysia	50% (6/12)	50% (3/6)
Total	54% (49/90)	86% (42/49)

SDF was taught in the undergraduate program in 86% (42/49) of the respondent schools, and half (21/42, 50%) of these schools had included SDF in the teaching for five or more years. Nearly half (19/42, 45%) of these schools allocated 1–2 h for the teaching of SDF while 17% of these schools allocated less time (Appendix S-Table 1).

**Fig. 1:**
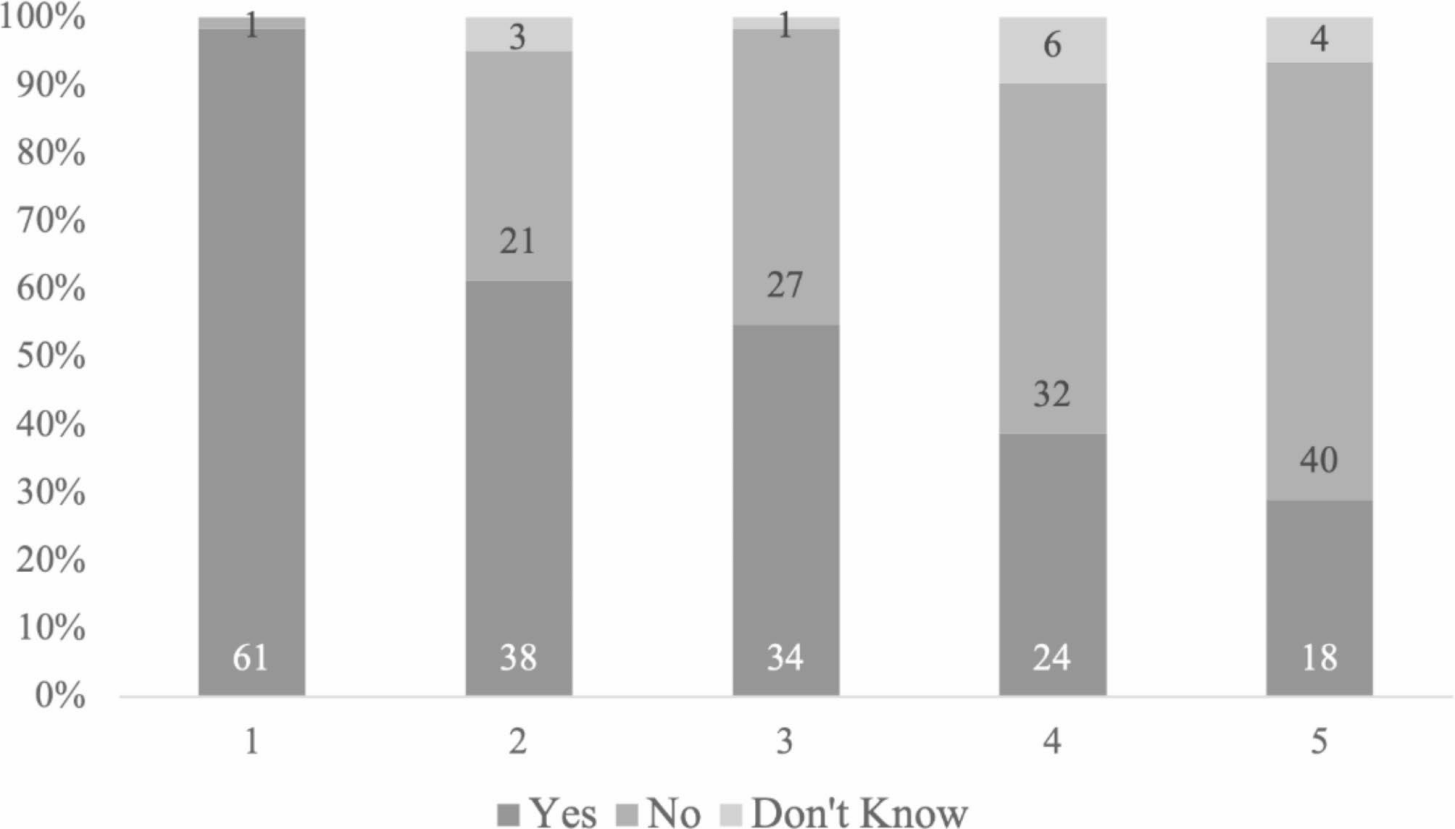
The proportion of departments according to the methods used in the teaching of SDF. (1: Lecture; 2 Case/group discussion by students 3: Clinical practice on humans (student/patients); 4: Seminar/workshop; 5: Simulation (practice on extracted/plastic teeth)). Figures in the bar refer to the number of departments

At the department level, 62 (77%) out of the 81 respondents covered SDF in its teaching program. Nearly all (61/62, 98%) used lecture in the teaching of SDF while 62% organized student group discussion (Fig. 1). Furthermore, 55% of the departments included SDF in its clinical teaching. The differences in the teaching of SDF between community dentistry and pediatric dentistry were not statistically significant (Data not shown).

### Content of the teaching

**Fig. 2:**
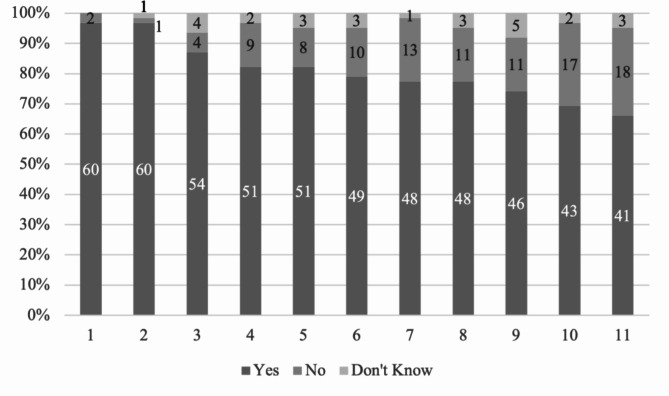
The proportion of departments according to the content of their teaching of SDF. (**1**: SDF can be used as an interim treatment in caries management; **2**: SDF can be used to arrest cavitated caries lesions in primary teeth; **3**: SDF can be used to arrest cavitated root surface caries in older adults; **4**: SDF can be used to arrest (prevent progression of) incipient noncavitated caries lesions in primary teeth; **5**: SDF can be used to arrest cavitated caries lesions in permanent teeth; **6**: SDF can be used to prevent root surface caries in older adults; **7**: SDF can be used to prevent caries in primary teeth of young children; **8**: SDF can be used to treat hypersensitive teeth; **9**: SDF can be used to arrest (prevent progression of) noncavitated root surface caries in older adults; **10**: SDF can be used to arrest (prevent progression of) incipient noncavitated caries lesions in permanent teeth; **11**: SDF can be used to prevent caries in permanent teeth; Figures in the bar refer to the number of departments)

There were no significant differences between the community dentistry and the pediatric dentistry departments regarding the indication contents of their teaching of SDF (all p values > 0.05) (Appendix S-Table 2). Nearly all (60/62, 97%) of the departments taught the use of SDF for arresting cavitated caries lesions in primary teeth (Fig. 2). Regarding the use of SDF to arrest noncavitated lesions (primary, permanent, and root caries), prevent caries (primary, permanent, and root caries) and treat dental hypersensitivity, the respective proportions were 69–82%, 66–79% and 77%. Table 2 shows that in the teaching, there were no significant differences in the indicated use of SDF for caries prevention and caries arrest in primary teeth and permanent teeth in children, and root surfaces in elderly (all p values > 0.05).

**Table 2:** Teaching contents of SDF in different tooth types

Teaching contents		Primary teeth N (%)	Permanent teeth N (%)	Root surface in the elderly N (%)	p-value^#^
**Prevent caries in**	Yes	48 (77)	41 (66)	49 (79)	0.367
	No	13 (21)	18 (29)	10 (16)	
	Don’t know	1 (2)	3 (5)	3 (5)	
**Arrest (prevent** **progression of)** **noncavitated caries in**	Yes	51 (82)	43 (69)	46 (74)	0.272
	No	9 (15)	17 (27)	11 (18)	
	Don’t know	2 (3)	2 (3)	5 (8)	
**Arrest -cavitated caries in**	Yes	60 (97)	51 (82)	54 (87)	0.068
	No	1 (2)	8 (13)	4 (7)	
	Don’t know	1 (2)	3 (5)	4 (7)	

^#^Chi-square test

Regarding the time of application, over half (35/62, 57%) of the respondents taught their undergraduate dental students to apply SDF for at least 60 s on the tooth surface. No significant differences in this teaching were detected between the two departments (Table 3).

Regarding the post-treatment instruction after SDF application, there was no dominant instruction (Table 3). One third (32%) of the respondents taught their students to ask the patient to refrain from eating and drinking for at least 30 min, followed by for at least 60 min (24%) and no need to refrain (21%). The differences in the teaching of post-treatment instructions between the pediatric dentistry and the community dentistry departments were statistically significant (p = 0.023).

SDF concentration of over 30% was most commonly adopted in the undergraduate teaching (Table 3). A higher proportion of the pediatric dentistry than community dentistry departments chose this concentration (P = 0.031).

**Table 3:** Concentration and time of SDF application and post-treatment instruction taught in different departments

		Community dentistry N (%)	Pediatric dentistry N (%)	Overall N (%)	p-value^#^
Concentration of SDF	Less than 20%	10 (30)	2 (7)	12 (19)	0.031*
	20–30%	7 (21)	4 (14)	11 (18)	
	More than 30%	16 (49)	23 (79)	39 (63)	
Time of application on teeth	Less than 10 s	5 (15)	1 (3)	6 (10)	0.327
	10–29 s	3 (9)	3 (10)	6 (10)	
	30–59 s	4 (12)	6 (21)	10 (16)	
	≥ 60 s	17 (52)	18 (62)	35 (57)	
	No standard application time	1 (3)	1 (3)	2 (3)	
	Not covered	3 (9)	0 (0)	3 (5)	
Time refrain from eating and drinking	Less than 30 min	4 (13)	1 (3)	5 (8)	0.023
	At least 30 min	9 (27)	11 (38)	20 (32)	
	At least 60 min	10 (30)	5 (17)	15 (24)	
	No need to refrain	7 (21)	6 (21)	13 (21)	
	No standard recommendation	0 (0)	6 (21)	6 (10)	
	Not covered	3 (9)	0 (0)	3 (5)	

**#**Chi-square test

### Reasons for not teaching SDF and plan

Seven dental schools did not teach SDF in any of the departments. At the department-level, nine pediatric dentistry departments and 10 community dentistry departments in the respondent dental schools did not include SDF in their undergraduate teaching program. The most common reason for not including SDF in the teaching was that SDF was unavailable in the department (10/19, 53%). Only one of these dental school did not plan to introduce SDF into their undergraduate teaching program (Table 4).

**Table 4:** Reasons for not teaching SDF and the plan to include SDF in the undergraduate teaching

		At school level (n = 7)	In community dentistry (n = 10)	In pediatric dentistry (n = 9)
Reasons for not teaching SDF^#^	Insufficient evidence to support SDF	0%	0%	22%
	No standard protocol	57%	30%	22%
	No available teacher/expertise	14%	20%	33%
	SDF is not available	71%	60%	44%
	SDF is too expensive	29%	40%	0%
	Potential adverse effects	14%	20%	44%
	Others	14%	20%	11%
Plan to teach SDF	No plan at present	14%	80%	33%
	Yes, in the coming 1–2 years	71%	10%	67%
	Yes, in the coming 3–4 years	14%	10%	0%

**#** Respondents could choose more than one answer for this question

## Discussion

In the present study, the online survey conducted a deep and detailed investigation of the extent to which SDF has been introduced into the undergraduate dental education in SEA. A total of 49 dental schools replied to this questionnaire survey. The responses came from almost all of the countries (except Laos) that provide dental undergraduate education in the SEA, which shows that the findings are representative to a certain extent. It is found that SDF is commonly included in the undergraduate programs in SEA. This is similar to the recently reported situation in the dental schools in the USA and in Canada [20–22]. And more common than those in the United Kingdom and in Iran [14, 16].

Around half of the respondent schools in the present survey introduced SDF into their undergraduate teaching in the past five years, which may be related to the recent international adoption of the SDF. The United States Food and Drug Administration (FDA) accepted the use of SDF to treat dentine hypersensitisation in 2014 and awarded breakthrough therapy status for approval of SDF as a drug to treat severe early childhood caries in 2017 [23].

In recent years, SDF has attracted increasing attention from researchers and practitioners from around the world. Previous studies claimed that dental schools are traditionally resistant to change [17]. However, this survey does not show such a phenomenon in relation to teaching SDF. Moreover, regarding the teaching method, half of the departments not only used didactic teaching but also included SDF in clinical practice teaching. The proportion of SEA dental schools with clinical practice applications is much higher than that in other countries reported recently [14, 16, 17, 20, 21]. The results indicate that dental schools in the SEA are more positive in adopting the clinical use of SDF. This may be related to the high prevalence of untreated caries among young children and the relatively low supply of dentists in the SEA countries.

SDF is a well-accepted method for arresting cavitated caries in young children. Almost all of the respondent dental departments in the present survey mentioned this in their undergraduate teaching. However, the application of SDF to arrest coronal caries in permanent teeth is less commonly covered in their undergraduate teaching, which is consistent with the finding of an earlier survey [20]. This may be because adults have a higher demand for restoration and aesthetics and prefer traditional treatments for decayed teeth. Whether SDF can be an effective agent for preventing coronal caries is still equivocal. Although laboratory studies have found that SDF can inhibit calcium dissolution from hydroxyapatite and prevent the demineralization of enamel and dentine, the related clinical evidence is still insufficient [24, 25].

Previous clinical trials have shown that SDF can be used to treat tooth hypersensitivity and can relieve pain and discomfort in adults [26, 27]. SDF was also first granted by the FDA for treating hypersensitivity. Nevertheless, nearly a quarter of the respondents in the present survey did not teach this application to their undergraduate students. Possibly because hypersensitivity has not attracted much attention and is easily overlooked the undergraduates teaching in the pediatric dentistry and community dentistry departments [28].

Regarding the application time, a review showed that most recommendations and studies preferred to apply SDF on teeth for at least 60 s to arrest caries. [29]. The present survey result is consistent in that more than half of the departments taught undergraduate students to apply SDF for at least 60 s.

The present study found that in the teaching of dental students in the SEA, there was no dominant instruction regarding refraining from eating and drinking after SDF application. In fact, evidence from clinical studies on this aspect is rare. This may be because, in the clinical protocols, dentists are requested to apply the SDF for a period time and also to isolate the tooth after SDF application for a while [29]. This may already provide adequate time for the SDF to exert its action on the carious lesion. Meanwhile, SDF is a water-based solution that can be easily washed away by saliva. There is little justification for the patients to refrain from eating and drinking after the application. Besides, some clinical protocols recommend placing a layer of sodium fluoride varnish on the SDF-treated lesion [29], even laboratory studies have not found a significant effect [30, 31], which may also be conducive to providing a circumstance for adequate exertion time.

Many products with different concentrations of SDF are available in the market, ranging from 3.8 to 38%, with 38% being the most popular [10]. It has been showed that 38% SDF is more effective than low concentrations in arresting caries [32]. The finding from the present survey is consistent with the current clinical evidence and most dental schools recommend the use of high concentration SDF.

In the present survey, the most commonly mentioned reason for not including SDF in the undergraduate teaching program is that SDF is unavailable in the dental school, followed by the potential adverse effects and lack of teachers or expertise. This finding is different from that of a previous survey on Canadian dental and dental hygiene programs whereby the most common barriers to cover SDF in the program were lack of consensus on clinical guidelines, and training and experience in using SDF [21].

There are some limitations in the present study. First, it was an online survey, and respondents replied by themselves instead of in a face-to-face interview. Thus, there were no chance for the respondents raise queries and no chance for us to clarify the answers from the respondents. Second, since the respondents had to put down the name of their school in the reply, they may provide more positive answers regarding the teaching of SDF than the actual situation so as to show that their school is more advanced in this aspect, leading to a biased result. Third, the respondents did not need to put down their own name and email address in the reply. So we could not resend an online questionnaire to the respondent to assess the reliability of their answers. Lastly, the present survey was conducted in only two departments but SDF might be taught in other specialties (e.g. cariology, restorative dentistry and geriatric dentistry.). The head of the department of pediatric dentistry and the head of community dentistry might not know the teaching of SDF in the other department. Therefore, the results of this study may have under-reported the teaching of SDF in the dental schools in South-east Asia.

## Conclusions

Results of the present online survey show that SDF has been widely taught in the dental schools in SEA. Using SDF to arrest cavitated caries lesions in primary teeth is commonly taught but the clinical protocol varies between schools. However, there were variations in the teaching of some potential applications of SDF, such as preventing caries and treating tooth hypersensitivity, among the departments. Developing a common syllabus and guidelines on the teaching of SDF to dental students and clinical practitioners in a more standard way is needed.

### Electronic supplementary material

Below is the link to the electronic supplementary material.

Supplementary Material 1

Supplementary Material 2

## Data Availability

The datasets used and/or analyzed during this study are available from the corresponding author upon reasonable request.
