# Supplementary material for: Teaching of silver diamine fluoride for the management of dental caries and hypersensitivity – situation in the Southeast Asia dental schools
Source: BMC Oral Health. 2023 Oct 29;23:815. doi: 10.1186/s12903-023-03502-0 (PMC10613390; doi:10.1186/s12903-023-03502-0)
Supplement: Supplementary file 1 — Supplementary Material 1 [file 12903_2023_3502_MOESM1_ESM.docx]

**Survey on teaching of SDF in the undergraduate programs of the dental schools in Southeast Asia**

**Consent to participate in the online survey**

- I have read and I understand the contents of the survey invitation email. I am willing to participate voluntarily in this survey.

**Part A - background information**

1. Which country is your university in?

____________________________

2. What is the name of your university?

____________________________

3. Which department/specialty do you represent?

- pediatric dentistry
- dental public health; community dentistry; preventive dentistry

**Part B – Teaching of SDF in your dental school**

4. As far as you know, is silver diamine fluoride (SDF) taught in the following courses in your dental school?

Yes No Don’t know

- Pediatric dentistry in undergraduate program
- Dental public health in undergraduate program
- Other clinical courses in undergraduate program
- Pediatric dentistry in postgraduate program
- Dental public health in postgraduate program
- Other clinical courses in postgraduate program

**The following questions are about the teaching of SDF in your department/specialty in the undergraduate program.**

5. Is SDF taught in your department/specialty in the **undergraduate** program?

- Yes 🡪 please continue to answer Questions 6-13
- No 🡪 please go to answer Questions 14-15

6. How long ago was the teaching of SDF introduced in the undergraduate program in your department/specialty?

- Less than 2 years
- 2-4 years ago
- 5-6 years ago
- More than 6 years ago

7. In the teaching on the use of SDF in the undergraduate program, are of the following methods used?

Yes No Don’t know

- Lecture
- Seminar/workshop
- Simulation (practice on extracted/plastic teeth/phantom heads)
- Clinical practice on humans (student/patients)
- Case/group discussion by students

8. In total, how many hours in the undergraduate program in your department/specialty are devoted to the teaching on SDF, i.e. all the timetabled activities？

- <1 hour
- 1-2 hours
- 3-5 hours
- >5 hours

9. In the undergraduate teaching regarding the use of SDF, is the following information delivered to the students?

Yes No Don’t know

- SDF can be used as an interim treatment in caries management.
- SDF can be used to prevent caries in primary teeth of young children.
- SDF can be used to prevent caries in permanent teeth.
- SDF can be used to prevent root surface caries in older adults.
- SDF can be used to arrest (prevent progression of) incipient non-cavitated caries lesions in primary teeth.
- SDF can be used to arrest (prevent progression of) incipient non-cavitated caries lesions in permanent teeth.
- SDF can be used to arrest (prevent progression of) non-cavitated root surface caries in older adults.
- SDF can be used to arrest cavitated caries lesions in primary teeth.
- SDF can be used to arrest cavitated caries lesions in permanent teeth.
- SDF can be used to arrest cavitated root surface caries in older adults.
- SDF can be used to treat hypersensitive teeth.
- SDF is indicated for use in patients (at any age) who are at high caries risk.
- SDF is indicated for use in young children who are uncooperative during dental treatment.
- SDF is indicated for use in patients (at any age) who are in special needs, e.g. with physical or mental impairment.
- SDF is indicated for use in patients (at any age) who are in poor general health, e.g. frail older adults.
- SDF is indicated for use in persons (at any age) who have great difficulties in accessing dental clinics for treatment.
- SDF is indicated for use in outreach dental services.
- There is no need to remove the surface dentine before applying SDF.

10. In each of the following clinical situations, which SDF application protocol is taught? (choose the main/most common protocol)

| One-off application | Multiple applications in a short time | Once a year | Twice a year | More than twice a year | Do not know |
| --- | --- | --- | --- | --- | --- |

- Prevention of caries
- Arrest initial non-cavitated caries
- Arrest cavitated caries
- Arrest root surface caries
- Treat hypersensitive teeth

11. According to the teaching in your department/specialty, which concentration of SDF is recommended?

- ＜20%
- 20-30%
- >30%

12. According to the teaching in your department/specialty, how long should SDF be applied on a tooth surface each time?

- Less than 10 seconds
- 10 to 29 seconds
- 30 to 59 seconds
- 1 minute or longer
- No standard application time
- Not covered in the teaching

13. According to the teaching in your department/specialty, for how long is a patient recommended to refrain from eating or drinking after SDF application?

- No need to refrain from eating or drink
- For less than 30 minutes
- For at least 30 minutes
- For at least 1 hour
- No standard recommendation
- Not covered in the teaching

**The following two questions are for the dental schools not including SDF in their undergraduate dental program**

14. What are the reasons why SDF is not covered in the undergraduate teaching in your department/specialty? (there can be more than one reason, select all and rank)

- SDF is not available in my country.
- SDF is not available in my dental school/clinic.
- Potential adverse effects of SDF (e.g. staining of tooth surface, irritation to soft tissues) are serious.
- No standard protocol for use of SDF is available.
- Insufficient evidence to prove that SDF is effective.
- Use of SDF is too expensive for the teaching programme.
- No teacher with the required knowledge and expertise is available.
- Other reasons, please specify: __________________

15. Does your department/specialty plan to introduce the teaching of SDF in the undergraduate program?

- No plan at present.
- Yes, in the coming 1-2 years.
- Yes, in the coming 3-4 years.
- Yes, probably later than 4 years

**End of questionnaire**

**Thank you for your participation.**
