# Supplementary material for: Teaching of silver diamine fluoride for the management of dental caries and hypersensitivity – situation in the Southeast Asia dental schools
Source: BMC Oral Health. 2023 Oct 29;23:815. doi: 10.1186/s12903-023-03502-0 (PMC10613390; doi:10.1186/s12903-023-03502-0)
Supplement: Supplementary file 2 — Supplementary Material 2 [file 12903_2023_3502_MOESM2_ESM.docx]

S-Table 1 Teaching of SDF in different departments

|  |  | **School level***  **N (%)** | **Community dentistry**  **N (%)** | **Pediatric dentistry**  **N (%)** | **P^#^** |
| --- | --- | --- | --- | --- | --- |
| **Introduced into teaching** | Less than 2 years ago | 8 (19) | 5 (15) | 8 (28) | 0.251 |
|  | 2-4 years ago | 13 (31) | 11 (33) | 11 (38) |  |
|  | 5-6 years ago | 11 (26) | 7 (21) | 7 (24) |  |
|  | More than 6 years ago | 10 (24) | 10 (30) | 3 (10) |  |
| **Hours devoted** | <1 hour | 7 (17) | 7 (21) | 13 (45) | 0.101 |
|  | 1-2 hours | 19 (45) | 20 (61) | 11 (38) |  |
|  | 3-5 hours | 10 (24) | 6 (18) | 4 (14) |  |
|  | >5 hours | 6 (14) | 0 (0) | 1 (3) |  |

^*^When there were two responses from two departments, the earliest time introduced SDF and the total hours devoted was taken and present in the school level column.

**^#^**Chi-square test

S-Table 2 Teaching contents of SDF between two departments

| **Teaching contents** |  | | | | | |
| --- | --- | --- | --- | --- | --- | --- |
|  |  |  | **Dental public health** | **Pediatric dentistry** | **Overall** | **P^#^** |
| **Indications —**  **SDF can be used** | interim treatment in caries  management | Yes | 32(97.0%) | 28(96.6%) | 60(96.8%) | 0.721 |
|  |  | No | 1(3.0%) | 1(3.4%) | 2(3.2%) |  |
|  | prevent caries in primary  teeth of young children | yes | 27(81.8%) | 21(72.4%) | 48(77.4%) | 0.349 |
|  |  | No | 5(15.2%) | 8(27.6%) | 13(21.0%) |  |
|  |  | Don’t know | 1(3.0%） | 0(0.0%) | 1(1.6%) |  |
|  | prevent caries in permanent teeth | Yes | 24(72.7%) | 17(58.6%) | 41(66.1%) | 0.486 |
|  |  | No | 8(24.2%) | 10(34.5%) | 18(29.0%) |  |
|  |  | Don’t know | 1(3.0%） | 2(6.9%) | 3(4.8%) |  |
|  | prevent root surface caries  in older adults | Yes | 27(81.8%) | 22(75.9%) | 49(79.0%) | 0.795 |
|  |  | No | 5(15.2%) | 5(17.2%) | 10(16.1%) |  |
|  |  | Don’t know | 1(3.0%) | 2(6.9%) | 3(4.8%) |  |
|  | treat hypersensitive teeth | Yes | 23(69.7%) | 25(86.2%) | 48(77.4%) | 0.215 |
|  |  | No | 7(21.2%) | 4(13.8%) | 11(17.7%) |  |
|  |  | Don’t know | 3(9.1%) | 0(0.0%) | 3(4.8%) |  |
|  | arrest (prevent progression of)  incipient non-cavitated  caries lesions in primary teeth | Yes | 27(81.8%) | 24(82.8%) | 51(82.3%) | 1.000 |
|  |  | No | 5(15.2%) | 4(13.8%) | 9(14.5%) |  |
|  |  | Don’t know | 1(3.0%) | 1(3.4%) | 2(3.2%) |  |
|  | arrest (prevent progression of)  incipient non-cavitated  caries lesions in permanent teeth | Yes | 23(69.7%) | 20(69.0%) | 43(69.4%) | 0.353 |
|  |  | No | 10(30.3%) | 7(24.1%) | 17(27.4%) |  |
|  |  | Don’t know | 0(0.0%) | 2(6.9%) | 2(3.2%) |  |
|  | arrest (prevent progression of)  non-cavitated root surface  caries in older adults | Yes | 24(72.7%) | 22(75.9%) | 46(74.2%) | 0.688 |
|  |  | No | 7(21.2%) | 4(13.8%) | 11(17.7%) |  |
|  |  | Don’t know | 2(6.1%) | 3(10.3%) | 5(8.1%) |  |
|  | arrest cavitated caries  lesions in primary teeth | Yes | 31(93.9%) | 29(100.0%) | 60(96.8%) | 1.000 |
|  |  | No | 1(3.0%) | 0(0.0%) | 1(1.6%) |  |
|  |  | Don’t know | 1(3.0%) | 0(0.0%) | 1(1.6%) |  |
|  | arrest cavitated caries  lesions in permanent teeth | Yes | 26(78.8%) | 25(86.2%) | 51(82.3%) | 0.441 |
|  |  | No | 6(18.2%) | 2(6.9%) | 8(12.9%) |  |
|  |  | Don’t know | 1(3.0%) | 2(6.9%) | 3(4.8%） |  |
|  | arrest cavitated root  caries in older adults | Yes | 28(84.8%) | 26(89.7%) | 54(87.1%) | 0.852 |
|  |  | No | 3(9.1%) | 1(3.4%) | 4(6.5%) |  |
|  |  | Don’t know | 2(6.1%) | 2(6.9%) | 4(6.5%) |  |
| **Indication-population** | patients (at any age)  who are at high caries risk | Yes | 25(75.8%) | 26(89.7%) | 51(82.3%) | 0.312 |
|  |  | No | 7(21.2%) | 3(10.3%) | 10(16.1%) |  |
|  |  | Don’t know | 1(3.0%) | 0(0.0%) | 1(1.6%) |  |
|  | young children who  are uncooperative  during dental treatment | Yes | 31(93.9%) | 28(96.6%) | 59(95.2%) | 1.000 |
|  |  | No | 1(3.0%) | 1(3.4%) | 2(3.2%) |  |
|  |  | Don’t know | 1(3.0%) | 0(0.0%) | 1(1.6%) |  |
|  | patients (at any age) who are  in special needs (e.g. with physical   or mental impairment) | Yes | 29(87.9%) | 29(100.0%) | 58(93.5%) | 0.073 |
|  |  | No | 4(12.1%) | 0(0.0%) | 4(6.5%) |  |
|  | patients (at any age) who are in poor general health  (e.g. frail older adults) | Yes | 27(81.8%) | 25(86.2%) | 52(83.9%) | 0.468 |
|  |  | No | 4(12.1%) | 1(3.4%) | 5(8.1%) |  |
|  |  | Don’t know | 2(6.1%) | 3(10.3%) | 5(8.1%) |  |
|  | persons (at any age) who  have great difficulties in accessing dental clinics for treatment | Yes | 28(84.8%) | 25(86.2%) | 53(85.5%) | 0.647 |
|  |  | No | 4(12.1%) | 2(6.9%) | 6(9.7%) |  |
|  |  | Don’t know | 1(3.0%) | 2(6.9%) | 3(4.8%) |  |
|  | outreach dental services | Yes | 31(93.9%) | 27(93.1%) | 58(93.5%) | 0.787 |
|  |  | No | 1(3.0%) | 2(6.9%) | 3(4.8%) |  |
|  |  | Don’t know | 1(3.0%) | 0(0.0%) | 1(1.6%) |  |
| **Preparation** | No need to remove the surface  dentine before applying SDF | Yes | 31(93.9%) | 28(96.6%) | 59(95.2%) | 1.000 |
|  |  | No | 1(3.0%) | 1(3.4%) | 2(3.2%) |  |
|  |  | Don’t know | 1(3.0%) | 0(0.0%) | 1(1.6%) |  |

**^#^**Chi-square test
